# The Pre-Analytical CEN/TS Standard for Microbiome Diagnostics—How Can Research and Development Benefit?

**DOI:** 10.3390/nu14091976

**Published:** 2022-05-09

**Authors:** Conny Stumptner, Vanessa Stadlbauer, Dominic O’Neil, André Gessner, Andreas Hiergeist, Kurt Zatloukal, Peter M. Abuja

**Affiliations:** 1 Diagnostic and Research Center for Molecular Biomedicine, Institute of Pathology, Medical University of Graz, 8010 Graz, Austria; cornelia.stumptner@medunigraz.at (C.S.); kurt.zatloukal@medunigraz.at (K.Z.); 2 Department of Internal Medicine, Division of Gastroenterology and Hepatology, Medical University of Graz, 8010 Graz, Austria; vanessa.stadlbauer@medunigraz.at; 3 Center of Biomarker Research CBMed, 8010 Graz, Austria; 4 QIAGEN GmbH, 40721 Hilden, Germany; dominic.oneil@qiagen.com; 5 Institute of Microbiology and Hygiene, 93053 Regensburg, Germany; andre.gessner@klinik.uni-regensburg.de (A.G.); andreas.hiergeist@klinik.uni-regensburg.de (A.H.)

**Keywords:** microbiome, diagnostics, European standard, in-vitro diagnostics, pre-analytics

## Abstract

Recently, CEN/TS 17626:2021, the European pre-analytical standard for human specimens intended for microbiome DNA analysis, was published. Although this standard relates to diagnostic procedures for microbiome analysis and is relevant for in vitro diagnostic (IVD) manufacturers and diagnostic laboratories, it also has implications for research and development (R&D). We present here why standards are needed in biomedical research, what pre-analytical standards can accomplish, and which elements of the pre-analytical workflow they cover. The benefits of standardization for the generation of FAIR (findable, accessible, interoperable, reusable) data and to support innovation are briefly discussed.

## 1. Introduction

In 2016, a survey among 1500 scientists [1] revealed that about 70% of them had failed to reproduce experiments or studies of others; this coined the term ‘reproducibility crisis’. In biomedical research, it was subsequently estimated that a large part of the failures was due to errors in the analysis of biological samples, specifically in the pre-analytical phase, indicating that the quality of the samples themselves was compromised [2,3]. This led to the development of European Technical Specifications and International Standards for sample pre-analytics (henceforth termed simply ‘standards’). They specify requirements regarding the pre-analytical procedure (workflow) and, most importantly, the documentation of information about variables and conditions along the entire pre-analytical workflow. However, they also allow sufficient freedom for researchers, clinicians, or companies to apply them according to their needs. The standards define requirements regarding the pre-analytical procedure and, most importantly, its documentation. The documentation of pre-analytical data facilitates later evaluation of whether a sample is fit for a particular purpose (i.e., whether it is suitable for a particular analytical test and for obtaining results that allow meeting the intended use of the analytical test). Fitness-for-purpose is of specific importance whenever analysis results/data should be reused in later research, which is an important part of the FAIR (findable, accessible, interoperable, reusable) data principle [4]. Standardization does not eliminate errors but helps to reduce them and enforces documented procedural quality. Moreover, compliance with standards is a prerequisite for reliable medical diagnostics and is essential for the development of diagnostic tests and quality monitoring schemes. Therefore, the EU In-vitro Diagnostics (IVD) Regulation 2017/746 (IVDR) [5], which defines requirements for diagnostic test developers and manufacturers to bring their tests to market, refers to such standards as state-of-the-art.

The need for standardization is increasingly recognized in the microbiome field. Over the last two decades, the field has rapidly evolved and become a matter of interest for science, industry and the public [6]. This was, in part, driven by the development of new technologies such as next-generation sequencing (NGS) and computational biology [7]. New discoveries not only led to an increased focus on the ways in which the microbiome interacts with the human body and thereby influences health and disease [8,9,10] but also to a large number of scientific publications [6]. Research funding for human microbiome research has grown dramatically, with over US 1.7 billion spent just during the past decade [11], and funding volumes for human microbiome studies increased to 920 million USD for 2012–2014 in the USA alone, which highlights the importance and timeliness of this research field [7,12]. The worldwide ’human microbiome market’ is flourishing and projected to grow by over 20% within the next few years (2025–2028) [13].

## 2. Need for Microbiome Standards in Clinical Practice and Diagnostics

Despite the rapid development of the microbiome field, several challenges have to be overcome to broadly implement microbiome diagnostics in clinical laboratories and apply them in routine medical practice. A major one is the above-mentioned ’reproducibility crisis’ in science that also affects the microbiome field [14]. Microbiome studies have often been difficult to reproduce and revealed inter-center variabilities [15]. Interlaboratory comparisons and proficiency testing have not been performed frequently; however, first published results indicate that the inter-platform variation is larger than the inter-laboratory variation, indicating that standardization processes can be successful [15,16,17]. Results of these interlaboratory comparisons organized by the German organization INSTAND e.V. show persistent methodological differences over the last seven years. Numerous factors and steps along the complex microbiome analysis workflow can affect the analysis result. They range from pre-analytical aspects such as patient/donor, specimen/sample, and metadata collection and processing, through molecular procedures such as biomolecule extraction from samples, quality analysis and sequencing, to bioinformatic processes such as assembly, annotation, integration of multiple omics data sets in computational analysis, visualizing and archiving [18,19,20,21]. Therefore, just like the analytical and post-analytical phases, the pre-analytical phase can lead to inaccurate and non-reproducible analysis/examination results that do not reflect the real situation in the human body but include variations induced by pre-analytical variables and errors.

Implementing a complex technology such as NGS into clinical diagnostics poses additional challenges. For a clinician, the result of a microbiome analysis has to be accurate; furthermore, it must usually be quantitative or at least semi-quantitative. It is also necessary to have reference values to be able to relate individual patients’ results to them. For this, understanding what a healthy microbiome looks like—a seemingly simple question that cannot be answered as easily as for less complex biomarkers—is of importance [22].

The large interest in the (human) microbiome for research, diagnostic and therapeutic applications on the one hand, and the difficulties in reproducing microbiome studies, on the other hand, led to calls for standards (i.e., guiding documents or norms) for microbiome research and diagnostics and for standardization of the microbiome analysis workflow [7,15,23,24]. Such a standard was published at the end of 2021: ‘CEN/TS 17626:2021 Molecular in vitro diagnostic examinations—Specifications for pre-examination processes for human specimen—Isolated microbiome DNA’ [25]. It is part of a series of international quality standards for sample pre-analytics developed by CEN and/or ISO in the context of the H2020 project SPIDIA4P. These normative documents are developed within the respective CEN and/or ISO working groups and subject to the directives of European/International standardization processes, ensuring the involvement and review of the respective National Standardization Bodies and their experts.

## 3. What Is a European/International Pre-Analytical Standard

According to CEN (https://www.cencenelec.eu/european-standardization/european-standards/#:~:text=The%20European%20Standards%20Bodies%20(CEN,degree%20of%20order%20in%20a, accessed on 25 March 2022) and ISO/IEC [26], a standard ‘is a document, established by consensus and approved by a recognized body, that provides, for common and repeated use, rules, guidelines or characteristics for activities or their results, aimed at the achievement of the optimum degree of order in a given context’. Further: ‘Standards should be based on the consolidated results of science, technology and experience, and aimed at the promotion of optimum community benefits’.

A European and international standard is a document that is developed by a group of experts that are part of larger groups called technical committees. Development of a new standard at ISO or CEN occurs in response to a request from stakeholders (e.g., potential users) (https://www.iso.org/developing-standards.html#:~:text=ISO%20standards%20are%20developed%20by,scope%2C%20key%20definitions%20and%20content, accessed on 25 March 2022). A standard is always agreed on at the European or international level involving a consultation procedure managed by non-profit organizations, such as the Comité Européen de Normalisation (CEN; European Committee for Standardization) and the International Standards Organization (ISO), respectively. Therefore, it is conceived by an agreement between standardization bodies and experts in the field to which the standard applies to ensure the inclusion of the current state-of-the-art. Consensus here means that the experts negotiate all aspects of the standard, including its scope, key definitions and content.

Standards should be based on the consolidated results of science, technology and experience and aimed at the promotion of optimum community benefits. They also provide guidance for their implementation and validation to guarantee compatibility, improve reproducibility and ensure quality. They are intended to lead to guaranteed (minimum) performance, interoperability, and fitness-for-purpose. Adherence to standards may be mandatory to comply with regulations that ensure performance, precision, security and safety, i.e., fitness-for-purpose. One such regulation is the IVDR in the EU.

Several standards, such as the pre-analytical standards, are/were developed first as CEN Technical Specifications (CEN/TS) and were or are then being adopted by the International Standards Organization (ISO) following the Vienna Agreement on Technical Co-Operation Between ISO and CEN (2001) (https://boss.cen.eu/media/CEN/ref/vienna_agreement.pdf, accessed on 25 March 2022). This ensures that European and International Standards are not in conflict with each other but are as consistent as possible with each other. Note that a Technical Specification (TS) serves as the normative document in those areas where the actual state-of-the-art is not yet sufficiently stable for a European Standard (European Norm, EN), or an agreement on an International Standard. A standard leads to full implementation, such as national standard, Europe-wide (CEN) or internationally (ISO), which may also serve regulatory purposes (https://boss.cen.eu/reference-material/Guidancedoc/Pages/Del; https://www.iso.org/deliverables-all.html, accessed on 25 March 2022). Here, we will not distinguish between ‘Standard’ and ‘Technical Specification’ but only use ‘standard(s)’.

Standards define requirements for products, processes and/or services to meet fitness-for-purpose. They are agreed on at the European or international level involving consultation procedures managed by non-profit organizations, such as CEN and ISO, respectively. A series of pre-analytical standards was developed in the context of the H2020 project SPIDIA4P, together with CEN, ISO and experts from their member countries.

Standards should not be mistaken for best practices or standard operating procedures (SOPs) because they differ widely in intent and specificity, as shown in Table 1.

### 3.1. Considerations Regarding a Diagnostic Pre-Analytic Standard for Isolated Human Microbiome DNA

Establishing a standard requires a prior definition of the terms it is meant to address. For CEN/TS 17626 this applied particularly to the term ‘microbiome’, which is very differently defined within the microbiome community [23]. Several groups define the microbiome as collective genomes and gene products of the ‘microbiota’ (i.e., the community of microorganisms) residing within a host or environment [9] (https://www.nature.com/subjects/microbiome, accessed on 25 March 2022). For the purpose of CEN/TS 17626:2021 it was chosen, in accordance with other groups, to define “microbiome” as the community of microorganisms in a well-defined habitat (environment), together with their biomolecules (such as RNA, DNA, proteins, lipids, polysaccharides, metabolites); it also comprises viruses, phages, plasmids and extracellular DNA, which are not considered as living microorganisms [23,25,27].

**Figure 1:**
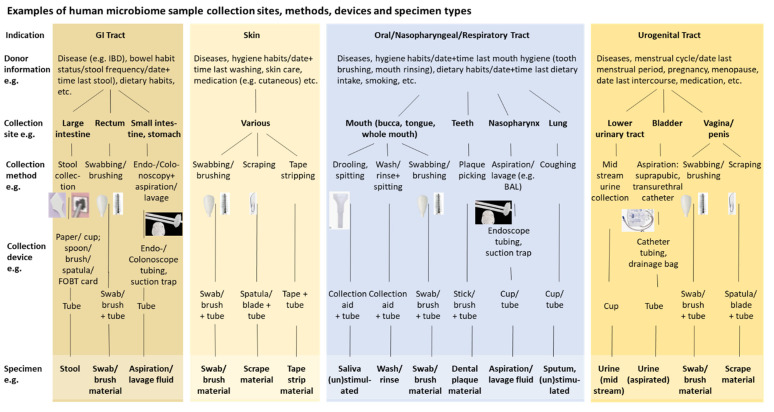
Examples of human microbiome sample types from different collection sites using different collection methods and devices. GI tract, skin, oral/nasopharyngeal/respiratory tract, and urogenital tract are major categories of anatomical sites from which samples for different indications and intended molecular examinations/analyses are collected in human diagnostics and R&D. This is associated with different pre-analytical variables, which include the patient/specimen donor, the exact topographical collection site in/on the body, and the collection method for which various collection devices exist, which finally leads to different types of human samples. CEN/TS 17626:2021 considers this and also reflects it in its structure.

Starting with the patient/specimen donor, a whole range of host factors, such as patient/donor age, diet, drug intake, ethnicity, geography, and lifestyle factors, have to be considered. With the collection, it needs to be defined where (collection site), how (collection method, devices, procedure and device application) and by whom (e.g., patient versus medical staff) a human specimen for microbiome DNA analysis is taken. The microbial composition, density and/or habitat differ depending on the body site and exact topographical region where the specimen is collected [28,29] (Figure 1). Duration and condition of specimen storage and transport before DNA isolation (i.e., with or without stabilization) can alter the microbiome profile as a consequence of ex-vivo growth or decline of certain microorganisms and/or degradation of microbial DNA [28,30]. Contamination with microbial and/or host cells and DNA can be unintentionally introduced to the specimen/sample during the pre-analytical workflow. Contamination may originate from the host cells at the collection site, depending on location and specimen/sample type [31]. Another source of contamination can be the chemicals or kits that are used during the pre-analytical phase (e.g., [32,33,34]). Contamination of samples with PCR amplicons requires spatial separation of pre-analytical steps from library preparation and sequencing. This is particularly critical with low microbial biomass specimens (e.g., skin, urine, bronchioalveolar lavage). Further critical pre-analytical variables and steps are the microbiome DNA isolation, including the method *per se,* the efficiency of the lysis of microbial cells, and interference and remaining content of inhibitory sample components (e.g., humic acids, polysaccharides) and human host DNA [28,35,36,37]. Implementation of appropriate controls at different steps of the pre-analytical workflow is of great importance. Comprehensive mixtures of microorganisms (mock communities) should be implemented and adapted to the respective sample material and scientific question. Buffer-only negative controls at random sample positions could support the identification of sources of contamination. Spike-in concepts should be implemented as process controls to identify the methodological bias for each individual sample and for absolute quantification of the detected species [38].

### 3.2. Structure of the Diagnostic Pre-Analytic Standard for Isolated Human Microbiome DNA

‘CEN/TS 17626:2021 Molecular in vitro diagnostic examinations—Specifications for the pre-examination processes for human specimen—Isolated microbiome DNA’ is a normative document that specifies requirements and gives recommendations for the pre-examination (i.e., pre-analytical) phase of human specimens, obtained from stool, saliva, skin and the urogenital tract, intended for examination of isolated microbiome DNA. Major target groups for this norm are medical laboratories, IVD developers and manufacturers, biomedical research institutions/organizations, biobanks, and regulatory authorities.

CEN/TS 17626:2021 belongs to a series of international standards for sample pre-analytics (Table 2), which share the same content structure. They are divided into two main sections—‘Outside the Laboratory’ and ‘Inside the Laboratory’ (Table 3). Note that the standards discriminate between ‘specimen’, which denotes the whole original material collected, and ‘sample’, which refers to an aliquot or part of the specimen.

‘Outside the Laboratory’ covers all steps from the patient/donor and the collection of (patient/donor) data and specimens to intermediate storage and transport to the laboratory, where further processing and possibly also biomolecular isolation of the specimen/samples are performed. Patient data are collected to specify factors that could alter the sample composition or might interfere with the analysis to clarify the type of analysis intended and to define the sample collection methods and devices (e.g., spitting of saliva, taking swabs, tape stripping, lavage). This is specified according to the collection site (skin, gastrointestinal, oral/nasopharyngeal/respiratory or urogenital tract). Steps outside the laboratory are typically performed by different persons in various facilities with varying degrees of certification or accreditation (e.g., collection by patients at home, at a physician’s office, in the hospital by medical personnel). These procedures are often not well standardized and documented. However, multiple sources of pre-analytical variation introduced at this stage can negatively impact sample quality and analysis results.

’Inside the Laboratory’ addresses pre-analytical workflow steps and variables starting with the arrival and subsequent processing of the sample in the laboratory (i.e., microbiome DNA isolation and determination of amount and quality), followed by storage until analysis. This section gives guidance regarding sample handling and documentation of pre-analytical variables such as those listed in Table 3. The standard discriminates between microbiome DNA isolation using commercial kits and laboratory developed/modified procedures and lists methods for quantity and quality assessment.

Similar to all documents of the pre-analytical standard series (Table 2), CEN/TS 17626:2021 refers to other relevant standards, such as the ISO accreditation standard for diagnostic laboratories, ISO 15189. They contain information the implementer must comply with to claim conformance with the pre-analytical standard. ISO 15189 relates to the entire diagnostic workflow, including analytical and post-analytical process steps, management and technical aspects. Conversely, the pre-analytical standard series addresses very specifically—and much more detailed than ISO 15189—the pre-analytical workflow for certain sample types (e.g., [39,40,41,42]). Therefore, the pre-analytical standards, including CEN/TS 17626:2021 are relevant for certified or accredited laboratories (e.g., ISO 15189) because they are considered state-of-the-art for pre-analytical sample handling and documentation by regulatory bodies and their auditors.

### 3.3. Benefit of Adhering to Standards in Research and Development

Academic and industrial microbiome R&D are presently not required to adhere to diagnostic pre-analytical standards for human biological samples unless this R&D will lead to diagnostic or therapeutic procedures, for which the IVDR applies. However, it is often advantageous to voluntarily comply with these standards since this can improve accuracy and reliability and supports the findability, accessibility, interoperability and reusability (FAIR) of results.

Standardization can increase cooperative research and contributes to solving the reproducibility crisis. Specifically, extensive annotation with pertinent metadata, as requested by all pre-analytical standards published so far, can vastly improve reusability since fitness-for-purpose can be assessed in a much better way. Moreover, in this way, the results generated in academic research can later be used to support certification and accreditation processes, e.g., according to IVDR.

### 3.4. Standards as Drivers of Innovation

Innovation is increasingly regarded as a process where information is freely shared for the benefit of the R&D community through what is often termed knowledge networks. However, the unrestricted introduction and uncritical use of information from various sources in such networks can be dangerous: a particular risk is here that information of insufficient reliability or quality may be used in a development process. An example is the well-known problem of financial losses caused by irreproducible clinical studies during drug development [43]. Another is the fact that misdiagnoses not only lead to enormous costs for health systems but were reported to cause 10% of patient deaths and 17% of adverse events [44]. Standards constitute a checkpoint against these problems since they enforce a basal level of procedural compliance, sufficient documentation, and reliability of results. In this way, they ensure present and future fitness-for-purpose, enable interoperability, and provide methods for avoiding, detecting and handling errors. Therefore, standards are a prerequisite for proficient knowledge sharing, in the sense of the FAIR principle. It is presently widely accepted that innovation flourishes when supported by standards since they help establish a solid, FAIR foundation as the basis for the evolution of methodologies. Standards and norms are indispensable for all industrial processes, services and products to ensure functional and safe products—and for the development of new ones.

## 4. Conclusions

Continuous efforts to standardize the entire workflow to achieve better reproducibility will be important for microbiome research and diagnostics. CEN/TS 17626:2021 makes an important first step in this direction—and should be adopted not only for diagnostics but for all microbiome-related R&D to generate a growing body of evidence based on data of comparable quality. Moreover, broad application of the standards will inevitably lead to broadening the scope of application and is indispensable for the development of improved methodologies and the creation of useful repositories of FAIR data.

## Figures and Tables

**Table 1:** Differences between Standards and Standard Operating Procedures (SOPs).

Pre-Analytical Standards (CEN/TS, ISO)	Standard Operating Procedures (SOP)
CEN/TS or ISO standards for sample pre-analytics are official documents that specify requirements (‘mandatory’; no deviation permitted; expressed as ‘shall’) and give recommendations (‘non-mandatory’; possible suitable choices, expressed as ‘should’) for the pre-analytical workflow of certain specimen types.	SOPs are written documents containing step-by-step instructions for laboratory procedures specific to a certain laboratory that the laboratory staff needs to follow.
They are evidence-based, written and agreed on by experts on a European (CEN) or international (ISO) level. They provide the state-of-the-art for national and international regulation (e.g., IVDR) and are considered by regulators to reduce the risk for IVD developers and users. They are applicable to a wider range of user-groups and are vendor neutral (i.e., they do not refer to specific products).	They are generated, reviewed, and approved by a particular laboratory. The information an SOP contains is more detailed and specific for the laboratory and its equipment, chemicals, reagents and procedure (e.g., defines the centrifugation speeds, temperature and duration).
CEN/TS and ISO pre-analytics standards provide the basis for SOPs.	SOPs are part of a laboratory’s quality management system and have to comply with requirements of standards.

CEN, European Committee for Standardization; ISO, International Standards Organization; SOP, Standard Operating Procedure; IVD, In-vitro diagnostics; IVDR, IVD regulation.

**Table 2:** Series of standards for sample pre-analytics as of March 2022.

ISO Standards and CEN Technical Specifications (CEN/TS) Molecular In Vitro Diagnostic Examinations—Specifications for Pre-Examination Processes for:
**Published**
EN ISO 20166-1: 2018, FFPE tissue—Part 1: Isolated RNA EN ISO 20166-2: 2018, FFPE tissue—Part 2: Isolated proteins EN ISO 20166-3: 2018, FFPE tissue—Part 3: Isolated DNA EN ISO 20166-4: 2021 FFPE tissues—Part 3: In-situ detection techniques EN ISO 20184-1: 2018, Frozen tissue—Part 1: Isolated RNA EN ISO 20184-2: 2018, Frozen tissue—Part 2: Isolated proteins EN ISO 20184-3: 2021, frozen tissue—Part 3: Isolated DNA EN ISO 20186-1: 2019, Venous whole blood—Part 1: Isolated cellular RNA EN ISO 20186-2: 2019, Venous whole blood—Part 2: Isolated genomic DNA EN ISO 20186-3: 2019, Venous whole blood—Part 3: Isolated circulating cell free DNA from plasma CEN/TS 17626: 2021, Human specimens—microbiome DNA CEN/TS 17390-1: 2020, Circulating tumor cells (CTCs)—Part 1: Isolated RNA CEN/TS 17390-2: 2020, Circulating tumor cells (CTCs)—Part 2: Isolated DNA CEN/TS 17390-3: 2020, Circulating tumor cells (CTCs)—Part 3: Preparation for analytical CTC staining EN ISO 23118: 2021, Urine, plasma, serum for metabolomics EN ISO 4307:2021, Saliva—Isolated human DNA CEN/TS 17688-1: 2021, Fine needle aspirates—Part 1: Isolated cellular RNA CEN/TS 17688-2: 2021, Fine needle aspirates—Part 2: Isolated proteins CEN/TS 17688-3: 2021, Fine needle aspirates—Part 3: Isolated genomic DNA
**Upcoming**
CEN/TS for exosomes and extracellular vesicles in venous whole blood—Isolated DNA /RNA/proteins CEN/TS for venous whole blood—Isolated circulating cell free RNA from plasma CEN/TS for urine and other body fluids—Isolated cell free DNA
Source: CEN/TC 140—In vitro diagnostic medical devices

All available CEN/ISO pre-analytical standards are listed here, and upcoming new standards are included.

**Table 3:** Structure of pre-analytical standards (based on CEN/TS 17626).

Structure (Major Chapters & Topics)		Topics or Examples of Pre-Analytical Factors Addressed
Introduction		Main information on microbiome (DNA) and the pre-analytical phase
1 Scope		Purpose/content: Requirements & recommendations for pre-examination phase of human specimens, such as stool, saliva, skin and urogenital specimens, intended for microbiome DNA examination Target group: Applicable to medical laboratories, in vitro diagnostics developers and manufacturers biobanks, biomedical research performing institutions/organizations, and regulatory authorities etc.
2 Normative references		Referral to other relevant standards:^1^ EN ISO 15189, ISO 15190, ISO/TS 20658
3 Terms and definitions		Definition of relevant terms
4 General considerations		Overarching information on relevance
5 Outside the laboratory	Patient/specimen donor	Demographics (e.g. age, gender, geography), disease/health condition, medication and treatment (incl. e.g. antibiotics), nutrition (incl. prebiotics, habits), frequency, life style, (smoking, personal care habits, stress, physical activity)
	Selection of specimen collection method & device(s) Specimen collection & stabilization	Collection method & device(s) (e.g. swab, tape, tube, collection site, spatula), process of collecting (incl. self-collection by donors), contamination, (pre-)treatment of collection site, labelling, stabilization (chemical, physical)
	Specimen storage & transport	Intermediate storage, with/without stabilizer, transport container, temperature, duration, oxygen, UV-light
6 Inside the laboratory	Specimen reception	Identification
	Sample preparation	Homogenization, enrichment (e.g. centrifugation), aliquoting, labelling
	Sample Storage	Duration, temperature, humidity, oxygen, UV-light
	Microbiome DNA isolation Quantity and quality assessment of microbiome DNA	Isolation method (e.g. cell lysis), reagents/kit (e.g. type, lot.no., contaminants), process of the isolation, host DNA content, quantity/quality assessment (e.g. method, process)
	Storage of microbiome DNA	Duration, temperature
Annex		Informative evidence-based information related to pre-analytical variables and the recommendations & requirements in the standard
Bibliography		

CEN/ISO pre-analytical standards follow the same general organization principle, presented using the diagnostic pre-analytical standard as an example.

## Data Availability

Not applicable.
